# Direct diagnostic testing of SARS-CoV-2 without the need for prior RNA extraction

**DOI:** 10.1038/s41598-021-81487-y

**Published:** 2021-01-28

**Authors:** Shan Wei, Esther Kohl, Alexandre Djandji, Stephanie Morgan, Susan Whittier, Mahesh Mansukhani, Eldad Hod, Mary D’Alton, Yousin Suh, Zev Williams

**Affiliations:** 1 Department of Obstetrics and Gynecology, Columbia University Medical Center, New York, USA; 2 Department of Pathology and Cell Biology, Columbia University Medical Center, New York, USA; 3 Department of Genetics and Development, Columbia University Medical Center, New York, USA

**Keywords:** Viral infection, Diagnostic markers

## Abstract

The COVID-19 pandemic has resulted in an urgent need for a rapid, point of care diagnostic testing that could be rapidly scaled on a worldwide level. We developed and tested a highly sensitive and robust assay based on reverse transcription loop mediated isothermal amplification (RT-LAMP) that uses readily available reagents and a simple heat block using contrived spike-in and actual clinical samples. RT-LAMP testing on RNA-spiked samples showed a limit of detection (LoD) of 2.5 copies/μl of viral transport media. RT-LAMP testing directly on clinical nasopharyngeal swab samples in viral transport media had an 85% positive percentage agreement (PPA) (17/20), and 100% negative percentage agreement (NPV) and delivered results in 30 min. Our optimized RT-LAMP based testing method is a scalable system that is sufficiently sensitive and robust to test for SARS-CoV-2 directly on clinical nasopharyngeal swab samples in viral transport media in 30 min at the point of care without the need for specialized or proprietary equipment or reagents. This cost-effective and efficient one-step testing method can be readily available for COVID-19 testing world-wide, especially in resource poor settings.

## Introduction

The Coronavirus disease 2019 (COVID-19) pandemic caused by SARS-coronavirus 2 (SARS-CoV-2) has created a global health emergency with more than three and a half million confirmed cases and more than 250,000 deaths as of May 5, 2020^1^. Widespread molecular diagnostic testing for the virus is crucial for prompt diagnosis and quarantine, treatment, and for a managed response to the ongoing SARS-Cov-2 pandemic^2^, especially given the apparently high rate of asymptomatic viral shedding^3^. The primary method for testing for active disease has been various forms of nucleic acid amplification tests (NAAT), including quantitative reverse-transcriptase polymerase chain reaction (RT-PCR)^4^. However, the majority of these assays require multiple steps including extended sample pretreatments, and/or RNA extraction and subsequent amplification and detection of specific regions of viral RNA, which can only be performed by shipping clinical samples to centralized high-complexity laboratories to perform the testing in batches^5–10^. The FDA has recently approved several simple and rapid molecular diagnostic tests for SARS-CoV-2, such as Abbott ID NOW and Cepheid’s Xpert Xpress, which can be performed at the point-of-care (POC). However, these systems rely on specialized, proprietary instruments and consumables which has contributed to the limited capacity to scale testing for widespread testing both in the U.S. and globally^11^. There is therefore a critical need for a SARS-CoV-2 diagnostic test that can be performed at the point-of-care with prompt delivery of results and without the need for costly and scarce proprietary equipment and reagents.

Loop-mediated isothermal amplification (LAMP) is a targeted nucleic acid amplification method that utilizes a combination of primer sets and a DNA polymerase with high strand displacement activity to specifically replicate a region of DNA^12^. Compared with traditional PCR, LAMP has several distinct advantages for point-of-care testing of clinical samples for SARS-CoV-2. While traditional PCR requires a costly and complex thermocycler, the entire LAMP amplification reaction is performed at a single temperature, and thus requires only a heat block or water bath. The polymerase used in LAMP (*Bst*) is more robust than that used in traditional PCR and can therefore function in the presence of PCR inhibitors frequently found in bodily fluids such as saliva and viral transport media (VTM), without the need to first purify the RNA. Because there is no need for thermocycling, DNA amplification is faster than with PCR and the product can be visualized with the unaided eye in real time using colorimetry, turbidity, or fluorescence with the aid of a fluorescent light source. The addition of a reverse transcription (RT) step (RT-LAMP) that occurs in the same master mix at the start of the reaction allows for detection of RNA targets, such as SARS-CoV-2. Finally, with a protocol that is readily available and public, compatible with a wide range of global suppliers for reagents, and that does not require specialized equipment, the method can be rapidly adopted, scaled, and iteratively improved. Thus, we sought to develop and share a highly-sensitive, one-step assay for SARS-CoV-2 that could be performed directly on clinical samples as a point-of-care test utilizing readily available reagents and equipment.

## Methods and materials

### RT-LAMP assay reagents preparation

Primer sets, buffers, and incubation methods were systematically tested to develop the optimized method used herein that would be sufficiently sensitive and robust to enable direct detection of viral RNA from clinical samples. PrimerExplorer V5 (https://primerexplorer.jp/e/) and the SARS-Cov-2 reference genome NC_045512v2 were used to design the LAMP primers. A 25-fold primer mix of LAMP primers (CUFC1-FIP, CUFC1-BIP, CUFC1-LF, CUFC-LB, CUFC1-F3, CUFC1-B3; Table 1, Supple Table 1) was prepared by assembling 40 µM FIP and BIP, 10 µM CUFC1-LF and CUFC1-LB, and 5 µM CUFC1-F3 and CUFC1-B3 primers in nuclease-free water (Ambion, AM9937). A 2X colorimetric RT-LAMP master mix was prepared by adding 3.5 µL 100 mM dUTP (Thermo Scientific, R0133), 0.5 µL Antarctic Thermolabile UDG (NEB, M0372S), and 0.25 µL 5 mM SYTO 9 (Invitrogen, S34854) to 1250 µL WarmStart Colorimetric LAMP 2X Master Mix (DNA & RNA) (NEB, M1800S/L). The reaction mix for one 250 µL reaction was prepared by mixing 125 µL 2X colorimetric RT-LAMP master mix, 10 µL 25-fold LAMP primer mix, and 95 µL nuclease-free water. These values can be scaled up according to the actual number of samples. Two 250 µL reactions were used to test one sample. Lysis buffer consisted of 0.1-fold buffer TE pH 8.0 (Ambion, AM9848) with 0.1% TWEEN-20, 1% volume (e.g., 1 µL enzyme added to 100µL buffer) Thermolabile Proteinase K (NEB, P8111S), 2% volume ezDNase (Invitrogen, 11766051), and 0.3 ng/µL human genomic DNA from a normal male. For one reaction, 460 µL of reaction mix and 20 µL of lysis buffer were preloaded in a clean 1.5 mL DNA LoBind microcentrifuge tube (Eppendorf, 022431021) and kept on ice until use.

**Table 1:** Sequence information of LAMP primers for SARS-Cov-2 detection.

Primer name	Sequence
CUFC1-F3	TGGATACAACTAGCTACAGAGAAG
CUFC1-B3	AGCCAAAGACCGTTAAGTGTA
CUFC1-FIP	GTGGTGGTTGGTAAAGAACATCAGACTTGTTGTCATCTCGCAAAGG
CUFC1-BIP	CCTCTATCACCTCAGCTGTTTTGCTGTACCATACAACCCTCAACTT
CUFC1-LF	ACCTGAGTTACTGAAGTCATTGAGA
CUFC1-LB	TGGTTTTAGAAAAATGGCATTCCC

### Initial testing of SARS-Cov-2 RNA-spiked and selected clinical samples

The limit of detection (LoD) was determined by testing serial dilutions in 1X HBSS (Gibco, 14025-092) of the SARS-Cov-2 RNA standard (Exact Diagnostics, COV019) 0.5 µL spike-in with 20 µL viral transport medium (VTM) (CDC SOP#: DSR-052-02), instead of clinical samples, following the optimized protocol detailed above. To determine the LoD, serial dilution was performed using 4–10 repeats. 95% CI is calculated using Clopper-Pearson method.

To determine the LoD with clinical samples, a set of 20 positive clinical samples was selected to represent the range of Ct values detected using a Roche cobas 6800 system for SARS-Cov-2 and 10 negative samples were subjected to the testing using the optimized LAMP protocol.

### Testing of clinical samples

A second set of 20 positive clinical samples consisting of viral transport media inoculated with a nasopharyngeal swab sample obtained as part of routine clinical testing was chosen at random. From each clinical specimen, 20 µL was placed directly into a 1.5 mL DNA LoBind microcentrifuge tube (Eppendorf, 022431021) containing the reaction mix (460µL) and lysis buffer (20 µL). The solution was mixed using a sterile disposal transfer pipette (Fisherbrand, 13-711-20) by gentle pipetting 12 times. Using the same sterile disposable transfer pipette, 250µL of the 500µL solution was placed into a new 1.5 mL DNA LoBind microcentrifuge tube. Both tubes with ~ 250 µL each were placed in a 63.0˚C dry bath (Fisherbrand, 14-955-219) and incubated for 30 min. The tubes were then placed on ice for 1 min to pause the reaction and the colorimetric results were read (red = negative, yellow = positive).

This study was reviewed and approved by the Institutional Review Board of Columbia University Irving Medical Center (CUIMC IRB) (Protocol# AAAS9910). The study used residual specimens from clinical care de-identified by indirect identifier. Consent was exempted for these samples by CUIMC IRB. All methods were carried out in accordance with relevant guidelines and regulations.

## Results

**Figure 1:**
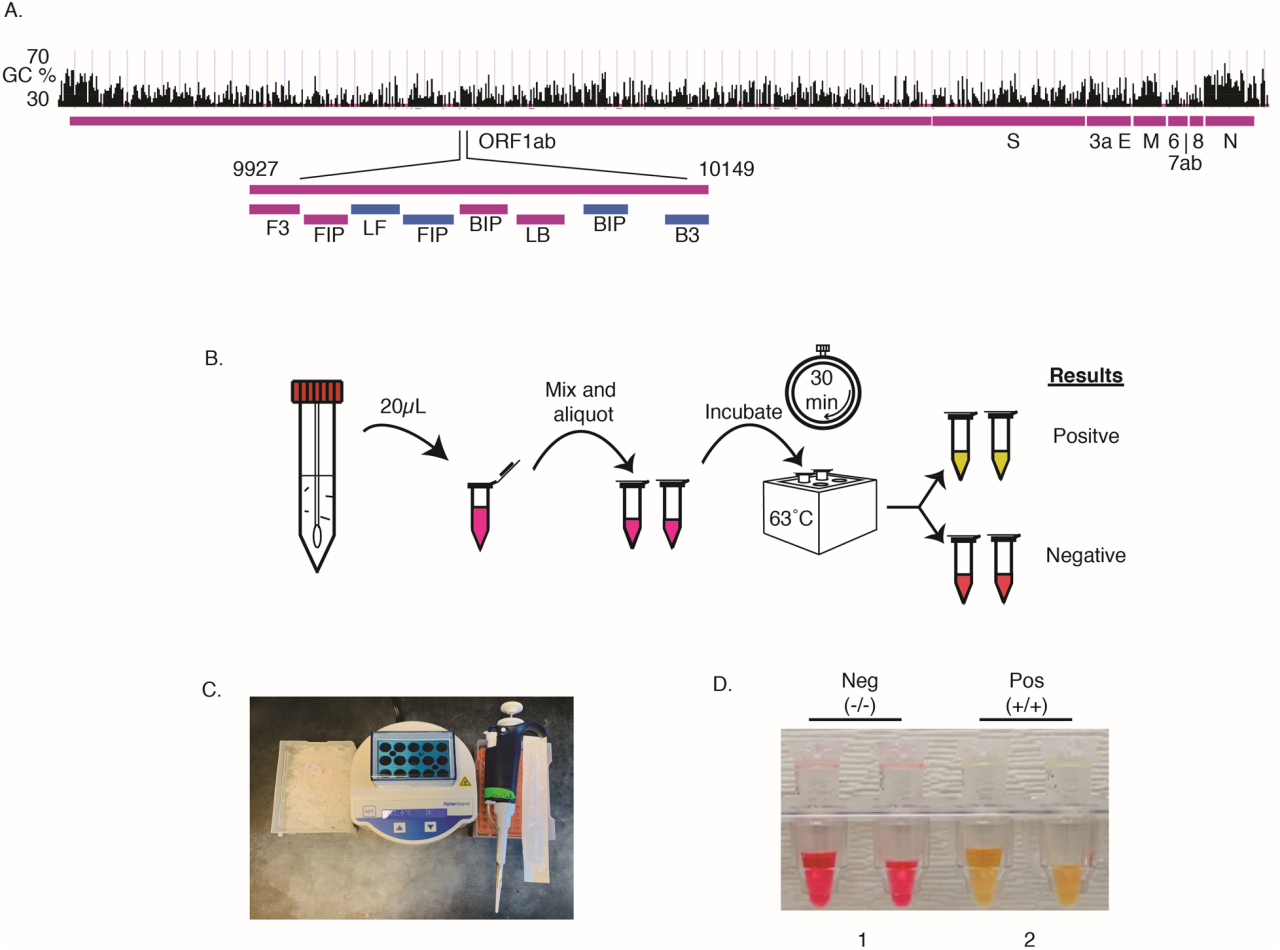
An RT-LAMP assay for rapid and direct SARS-Cov-2 testing of clinical sample and the point-of-care. (**A**) Primer design. A set of 6 LAMP primers targeting the middle of the Orf1ab gene. Sequences and primers matching to the + strand of virus genome are in pink, while those matching to the—strand are shown in blue. Illustration of GC% of SARS-CoV-2 genome was from UCSC genome browser (http://genome.ucsc.edu)^17,18^. (**B**) Workflow of direct RT-LAMP assay. 20 µL of raw clinical samples from nasal swab in viral transport media (VTM) was added to 480 µL reaction solution consisting of reaction master mix and lysis buffer, mixed and 250 µL were aliquoted with a transfer pipette and then incubated on heatblock for 30 min. The reaction was stopped by placing the samples on ice and results were interpreted by color due to a pH sensitive dye in the master mix (yellow = positive; red = negative). (**C**). Setup of the RT-LAMP assay. (**D**). An example of colorimetric results on negative and positive clinical samples.

We designed and optimized LAMP primers and reaction conditions for high performance, direct rapid colorimetric RT-LAMP testing for SARS-CoV-2 (Fig. 1). The final primer set targeted the middle of *ORF1ab,* the largest SARS-CoV-2 gene (Fig. 1A), and has relatively low GC%. The optimal reaction temperature was determined experimentally and set to be 63˚C. The workflow enables direct testing of clinical samples without the need for RNA isolation or cell lysis (Fig. 1B)^6,13^. The set-up requires only a pipette and tips, a transfer pipette, a mini heat block, and a box of ice (Fig. 1C); no special equipment or devices are needed. We used a colorimetric output for simple interpretation of results without the need for extra equipment. RT-LAMP amplification of the targeted DNA results in decreased pH. A pH sensitive dye added to the reaction mixture resulted in a color change from red to yellow in a positive test and remained red in negative tests (Fig. 1D). For samples with low copy number of virus, a positive signal was displayed only in one of the two tubes, in which case we considered the results as positive.

**Figure 2:**
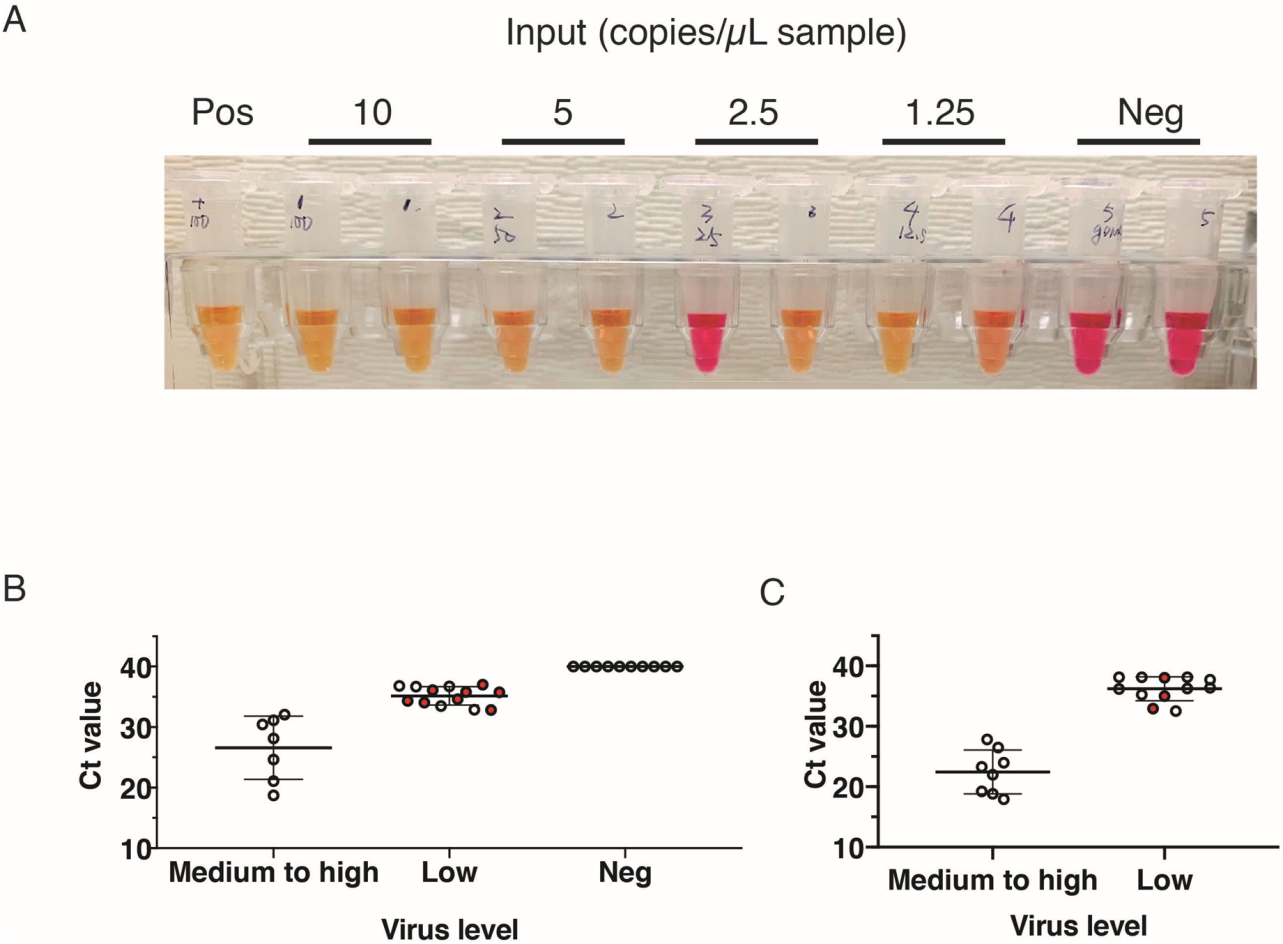
The performance of RT-LAMP testing for SARS-Cov-2. (**A**) Estimation of limit of detection using VTM and RNA standard spike-ins. (Colorimetric results: red = negative; yellow = positive). (**B**) Estimation of limit of detection using clinical samples selected to represent a wide range of Ct values. Each dot represents the Ct value of target 2 of one sample, and a sample with discordant testing result is labeled in red. Error bar indicates Mean ± SD. (**C**) Testing on randomly selected positive and negative clinical samples. Each dot represents the Ct value of target 2 of one sample, and a sample with discordant testing result is labeled in red. Error bars indicates mean ± SD.

Viral transport medium contains inhibitors that reduce sensitivity of amplification and we found that it reduced the sensitivity of our RT-LAMP reaction by 30 to 100-fold compared with buffers such as HBSS^6^. Nonetheless, we continued to use samples collected as part of clinical care that had been placed in viral transport media so that we could (a) keep the existing workflow as consistent as possible and (b) have a single nasopharyngeal swab sample tested in parallel using our test and the Roche cobas system. To determine the LoD of our assay, we spiked-in viral RNA standards into viral transport media (Fig. 2A). Serial dilution experiments, conducted in quadruplicate, consistently showed positive results down to 2.5 copies/µL. Results with copy number below 2.5 copies/µL were inconsistent, and thus we determined 2.5 copies/µL to be our LoD (Fig. 2A).

To compare the Ct values of clinical samples tested on the Roche cobas system with our RT-LAMP results, positive clinical samples from routine clinical testing for COVID-19 were selected to represent the broad range of Ct value and tested using our LAMP assay (Fig. 2B). These samples showed Ct values ranging from 18.52 to 34.42 for RT-PCR Target 1, and 18.69 to 36.61 for RT-PCR Target 2 on the Roche cobas 6800 system. Ten negative clinical samples were also tested. Samples with Ct value of ≤ 30 for target 1 and ≤ 31 for target 2, corresponding to ~ 0.168–0.252 TCID_50_/mL^14^, had stable performance and high accuracy in testing results (Fig. 2B). Hence the LoD of clinical samples using our RT-LAMP assay is Ct ≤ 30 for RT-PCR target 1 and ≤ 31 for RT-PCR target 2. Five out of 13 samples with high Ct values tested positive, and all of the false negative samples were not necessarily the ones with highest Ct value, indicating that the presence of detectable virus was not within the linear range due to low copy numbers. All of the 10 negative samples were negative by RT-LAMP, indicating that it had a specificity of 100% (binomial 97.5% unidirectional confidence limit 69.2–100%) (Fig. 1B, Table 2).

**Table 2:** Summary of direct RT-LAMP testing on clinical samples.

Sample	Number of sample (n)	Concordant with Roche cobas 6800	PPA (%; n)	NPA (%; n)
Medium to high (≥ LoD)	8	8	100 (8/8)	–
Low (< LoD)	12	9	75 (9/12)	–
Neg	10	10	–	100 (10/10)
Total	30	27	85 (17/20)	100 (10/10)

*PPA* positive percentage agreement, *NPA* negative percentage agreement.

To estimate the detection power of our RT-LAMP test on actual clinical specimens, a second set of 20 clinical samples tested positive and 10 clinical samples tested negative by standard test were randomly selected and tested. Samples had a Ct value ranging from 17.46 to 35.71 for Target 1, and 17.94 to 38.12 for Target 2. Eight samples were within, while 12 samples were below, the predicted LoD of the rapid testing method (Fig. 2C). Eight out of 8 samples within LoD were tested positive and were in concordance with clinical testing results. Nine out of 12 samples below LoD were tested positive. Taken together, our results indicate that RT-LAMP has the 75% (9/12) positive percentage agreement (PPA) below LoD, 100% (8/8) PPA within LoD, and 100% (10/10) negative percentage agreement (NPA) (Table 2). Together, the rapid testing generated an 85% (17/20) PPA, 100% (10/10) NPA, and 90% (27/30) accuracy for randomly selected clinical samples.

## Discussion

Here we developed a robust and highly sensitive method based on RT-LAMP for direct detection of SARS-CoV-2 in viral transport media in 30 min with a LoD as low as 2.5 copies/μl. A unique feature of our assay is that it does not require RNA isolation and/or cell lysis, and could be applied directly to clinical samples, unlike other recent reports using RT-LAMP for SARS-CoV-2 test^13,15^. The ability to test at the point-of-care and return results within 30 min without the need for RNA extraction/purification or specialized equipment has practical advantages for on-site screening and detection of those with a higher viral load. In particular, the RT-LAMP test may be useful as a primary screening to provide a quick diagnostic for patients at the early stage of spreading and without significant symptoms, when the patients are normally reported to have 10^4^ to 10^7^ copies/mL virus load^16^. Thus, this method would also lend itself to widespread testing and testing in resource-poor settings. Additionally, RT-LAMP has been shown to be compatible with different biological fluids (e.g. saliva, urine). While our results were highly specific with no false positive results observed, our assay was not as sensitive as the Roche cobas 6800 system. Further optimization in primer design, adjustments to buffers, multiplex primers targeting multiple regions of the virus, and other adjustments, improving the sensitivity of the assay as well as validation would be required to improve the assay. Viral transport media contains inhibitors that reduced the sensitivity of our assay by ~ 30 ×, we still chose to use nasopharyngeal swab samples placed in viral transport media for this study so that the testing could be readily incorporated into the existing testing workflow and so that a single sample could be processed using our method and the Roche cobas 6800 system for validation. However, for use as a point-of-care test where transport is not needed, using a more basic buffer such as HBSS or direct placement of the swab into the master mix reaction buffer could make this processes simpler, faster and more sensitive. The use of saliva directly could also potentially increase sensitivity, both by avoiding inhibitory VTM and using a sample that may have higher clinical sensitivity^16^. It should also be noted that the PPV and NPV will depend on the prevalence of infection at the location where the testing is performed.

To successfully manage the COVID-19 outbreak, cost-effective, efficient, and frequent screen is necessary world-wide. The speed, ease-of-us, and scalability of this readily available RT-LAMP method and widely-sourced reagents make it well suited for an initial screening at the point of care and at population levels.

## Supplementary Information

Supplementary Information.
