# Supplementary material for: Direct diagnostic testing of SARS-CoV-2 without the need for prior RNA extraction
Source: Sci Rep. 2021 Jan 28;11:2402. doi: 10.1038/s41598-021-81487-y (PMC7844049; doi:10.1038/s41598-021-81487-y)
Supplement: Supplementary file 1 — Supplementary Information. [file 41598_2021_81487_MOESM1_ESM.docx]

**Supplemental information**

**Direct diagnostic testing of SARS-CoV-2 without the need for prior RNA extraction**

Shan Wei^1^, Esther Kohl^1^, Alexandre Djandji^1^, Stephanie Morgan^1^, Susan Whittier^2^, Mahesh Mansukhani^2^, Eldad Hod^2^, Mary D’Alton^1^, Yousin Suh^1,3^, and Zev Williams^1^ *

^1^Department of Obstetrics and Gynecology, Columbia University Medical Center, New York, USA

^2^Department of Pathology and Cell Biology, Columbia University Medical Center, New York, USA

^3^Department of Genetics and Development, Columbia University Medical Center, New York, USA

* Corresponding author

[zw2421@cumc.columbia.edu](mailto:zw2421@cumc.columbia.edu)

**Supplemental Table 1. In silico cross-reactivity analysis**

******
